# Accuracy and feasibility with AI-assisted OCT in retinal disorder community screening

**DOI:** 10.3389/fcell.2022.1053483

**Published:** 2022-11-03

**Authors:** Jianhao Bai, Zhongqi Wan, Ping Li, Lei Chen, Jingcheng Wang, Yu Fan, Xinjian Chen, Qing Peng, Peng Gao

**Affiliations:** ^1^ Department of Ophthalmology, Shanghai Tenth People’s Hospital of Tongji University, Tongji University School of Medicine, Shanghai, China; ^2^ Suzhou Big Vision Medical Technology Co Ltd, Suzhou, China; ^3^ School of Electronic and Information Engineering, Soochow University, Suzhou, China

**Keywords:** artificial intelligence (AI), optical coherence tomography (OCT), retinal disorders, community ophthalmic screening, accuracy

## Abstract

**Objective:** To evaluate the accuracy and feasibility of the auto-detection of 15 retinal disorders with artificial intelligence (AI)-assisted optical coherence tomography (OCT) in community screening.

**Methods:** A total of 954 eyes of 477 subjects from four local communities were enrolled in this study from September to December 2021. They received OCT scans covering an area of 12 mm × 9 mm at the posterior pole retina involving the macular and optic disc, as well as other ophthalmic examinations performed using their demographic information recorded. The OCT images were analyzed using integrated software with the previously established algorithm based on the deep-learning method and trained to detect 15 kinds of retinal disorders, namely, pigment epithelial detachment (PED), posterior vitreous detachment (PVD), epiretinal membranes (ERMs), sub-retinal fluid (SRF), choroidal neovascularization (CNV), drusen, retinoschisis, cystoid macular edema (CME), exudation, macular hole (MH), retinal detachment (RD), ellipsoid zone disruption, focal choroidal excavation (FCE), choroid atrophy, and retinal hemorrhage. Meanwhile, the diagnosis was also generated from three groups of individual ophthalmologists (group of retina specialists, senior ophthalmologists, and junior ophthalmologists) and compared with those by the AI. The area under the receiver operating characteristic curve (AUC), sensitivity, and specificity were calculated, and kappa statistics were performed.

**Results:** A total of 878 eyes were finally enrolled, with 76 excluded due to poor image quality. In the detection of 15 retinal disorders, the ROC curve comparison between AI and professors’ presented relatively large AUC (0.891–0.997), high sensitivity (87.65–100%), and high specificity (80.12–99.41%). Among the ROC curve comparisons with those by the retina specialists, AI was the closest one to the professors’ compared to senior and junior ophthalmologists (*p* < 0.05).

**Conclusion:** AI-assisted OCT is highly accurate, sensitive, and specific in auto-detection of 15 kinds of retinal disorders, certifying its feasibility and effectiveness in community ophthalmic screening.

## Introduction

With the rapid progress in population aging and the escalating prevalence of systemic diseases like hypertension and diabetes mellitus, as well as the increasing incidence of myopia in contemporary society, the morbidity rate of multiple ophthalmic diseases, especially with various retinal disorders, has ascended consequently. It caused visual impairment and even blindness in both developed and developing countries (Hong et al., 2013; Wolfram et al., 2019; Li et al., 2022b). Among them, the age-related macular degeneration (AMD), diabetic retinopathy (DR), and myopic retinopathy, as well as other macular disorders like epiretinal membranes (ERMs) and macular holes, were the significant components and chief culprits for visual loss in most populations (Mitchell et al., 2018; Wang and Lo, 2018; Ruiz-Medrano et al., 2019).

Early diagnosis and prompt treatment were essential to achieve the best possible visual prognosis (Mitchell et al., 2018; Wang and Lo, 2018; Ruiz-Medrano et al., 2019). Therefore, early detection at the initial stages and positive screening at the pre-hospital level were necessary, indicating the importance of effective and efficient community screening. A notable and challenging fact exists on the insufficient medical human resources allocated in the community and the inadequately experienced ophthalmologists in primary hospitals, forging a considerable gap and actual bottleneck toward the enormous demand for community screening (Feng et al., 2018).

With tremendous progress in recent years, artificial intelligence (AI) has been integrated with various fields of science and taken to practical engineering in multiple scenes. The deep-learning (DL) algorithm is an advanced type of machine learning (ML) with a multi-layered convolutional neural network (CNN) model. It is capable of learning and detecting image features and recognizing patterns from a large dataset. The application of DL in medicine has fulfilled the function of automated lesion recognition and prognosis prediction in various diseases (Gulshan et al., 2016; Zhang Q. et al., 2021). In the field of ophthalmology, different AI algorithms have been developed and applied for auto-detection of diverse diseases like glaucoma (Keel et al., 2019; Camara et al., 2022), ocular surface diseases (Zhang Y. Y. et al., 2021), and multiple retinal disorders, like DR, AMD, and myopic retinopathy, and have exhibited relatively high accuracy and reliable performance in clinical diagnosis as well as the pre-hospital community screening, providing a promising solution for the challenge mentioned previously (Rajalakshmi et al., 2018; He et al., 2020; Xie et al., 2020; Cen et al., 2021).

One disadvantage of AI algorithms trained to detect retinal disorders is that they are mainly based on the features from fundus photography images, which could not offer a comprehensive message of deep layers of the retina and the choroid (He et al., 2020; Xie et al., 2020; Cen et al., 2021). Meanwhile, the optical coherence tomography (OCT) technique can provide the cross-sectional images of the retina with high resolution realizing *in vivo* visualization of cellular tissue microstructure *via* interferometry and has been widely utilized in the clinical practice of ophthalmology. By providing the morphological features and quantitative measurement data of the different layers of the retina, especially at the macular and around the optic head, and the certain depth of the choroid, OCT shows its advantage in detecting multiple retinal diseases superior to utilizing fundus image only. One emerging trend is to develop AI algorithms based on OCT images to diagnose some retinal diseases. At the same time, several studies have shown its feasibility and revealed its accuracy in clinical application (Sandhu et al., 2018; Treder et al., 2018; Sandhu et al., 2020; Sogawa et al., 2020; Wang et al., 2020).

Another major disadvantage of current AI software was that it mostly focuses only on one specific retinal disorder. In comparison, a very considerable proportion of patients suffer from more than one retinal disorder, which redistricts the practical application of this AI software in a real clinical setting (Sandhu et al., 2018; Treder et al., 2018; Sandhu et al., 2020; Sogawa et al., 2020; Wang et al., 2020). Therefore, the impending demand rising from clinical practice and community screening work is to develop AI algorithms that can recognize multiple retinal disorders simultaneously in single detection.

In view of the aforementioned requirement, this study involved the utilization of an AI-assisted OCT instrument to examine the retina in a community screening, with the acquired images analyzed using a pre-designed DL algorithm that could detect 15 retinal disorders simultaneously, aiming to evaluate its accuracy and feasibility in the practical application.

## Materials and methods

### Participants’ enrollment

The inhabitants of four local communities (DaNing Road community, GongHe Road community, PengPu Town community, and Linfen Road community, all of which are located in Jing’an District, Shanghai) participated in the study. The study period was from September to December 2021.

The inclusion criteria were: 1) age>18 years; 2) co-operation with the ophthalmic examinations; and 3) voluntary participation in the study. The exclusion criteria were as follows: 1) patients with ophthalmic disease causing severe refractive media opacity like keratoleukoma, cataract, and vitreous or within ophthalmic emergency situations; 2) patients with other severe uncontrolled systemic diseases; and 3) patients during pregnancy or lactation period.

The study was approved by the Ethical Committee of Shanghai Tenth People’s Hospital and conducted in accordance with the tenets of the Declaration of Helsinki, with the written informed consent forms signed by all the participants voluntarily.

#### Community screening

All the participants underwent the following ophthalmic examinations: 1) best-corrected visual acuity (BCVA); 2) intraocular pressure (IOP), performed using an iCare tonometer device (iCare IC 100, Icare Oy, Vantaa, Finland); 3) slit-lamp examination (YZ5X, Suzhou 66 Vision, Suzhou, China); 4) automatic nonmydriatic fundus photography (TRC-NW400, Topcon, Tokyo, Japan), images captured with both macula-centered and disc-centered; and 5) spectral domain-OCT scan (SD-OCT, BV1000, Bigvision Inc., Jiangxi, China). Meanwhile, the demographic information of the participants and their general medical history of systemic diseases, such as hypertension and diabetes mellitus, were recorded.

The OCT scan covered an area of 12 mm × 9 mm at the posterior pole of the retina involving the macular and optic disc in one image view and extended to a depth of 2.3mm, with the axial resolution of 5 μm and the horizontal resolution of 20 μm in tissues. Owing to the maximum A-scan speed at 45,000 times per second and the automated voice prompt operating system, the process of the examination was convenient and fast. The images were then transmitted to the AI algorithm, which was integrated into the instrument to generate an analysis of multiple retinal disorders. During the process, the time duration for the OCT examination for one eye (starting from the participant placing their head and chin at the bracket to terminating at the end of the scan for one eye) and the output time of the AI report were also recorded.

### AI auto-detection

**FIGURE 1:**
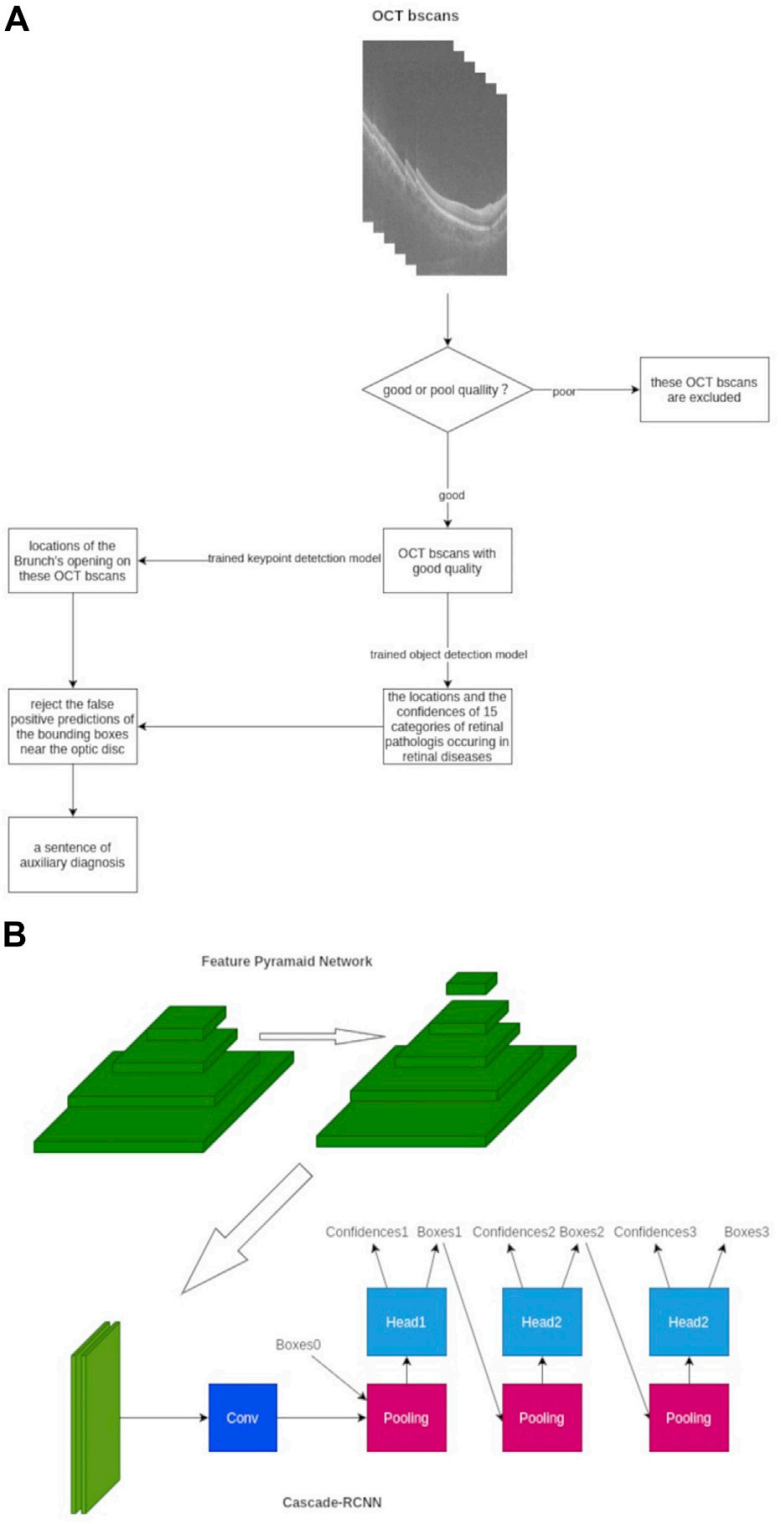
Framework of our AI algorithms designed to detect 15 different retinal disorders. **(A)** Workflow chart of AI algorithm **(B)** Framework architecture and model establishment of our AI algorithm with FPN-Cascade-RCNN. Conv, convolution.

The inference pipeline was as follows: first, the quality of collected OCT images was assessed using an AI algorithm. The OCT b-scans with poor quality, such as heavy noise, severe artifacts, low contrast, or low brightness, were excluded to avoid the analysis results with low confidence. The module of quality assessment is a trained binary classification model, which is composed of ResNet-50. Then, the OCT b-scans with good quality were imputed into a trained object detection model to detect up to 15 categories of retinal pathologies, and the detection model was based on the deep-learning convolutional neural network. Subsequently, the trained key-point detection model extracted the Bruch’s membrane opening locations on OCT b-scans, and the false optimistic predictions of the bounding boxes near the site of the optic disc would be eliminated. Finally, a probability ratio (0–1) of auxiliary diagnosis based on all considerations and lesion locations of 15 categories of retinal disorders on OCT b-scans was calculated and applied (Figure 1).

As for the development strategy of the AI algorithm, a multi-stage object detection model based on the adjusted Cascade-RCNN was adopted (Essa et al., 2011). The feature pyramid network architecture was used to extract the multi-scale features of each b-scan image to enhance the ability of the model to detect large-size retinal and tiny-size pathologist such as retinal detachment and exudation. We trained the model for 48 epochs, and the SGD optimizer was used (Zhang Z. et al., 2021). The Smooth L1 loss function was used in the regression of the region proposal network and cascade-head network, and the cross-entropy loss was used in classification parts. The online hard example mining mechanism was used to improve the convergence speed due to the unbalanced ratio between the foreground and background, and the tremendous differences in learning difficulty among 15 categories of retinal disorders were observed (Chen et al., 2021). As for the datasets, 1311 cubes were collected and divided into the training set, validation set, and test set with a ratio of 6:2:2. The distribution of the datasets is presented in Supplementary Table 1, which shows 363 cubes are normal, and most of the retinal disorders are PVD. Three or more typical b-scans slices were selected from cubes, and these selected slices contained various lesions of retinal disorders, which contributed to the better learning and adaptive ability of AI algorithm. Subsequently, the external test was performed, and the results of the external test set can be used as evidence of the generalization ability of the AI model.

**TABLE 1:** Demographic information and baseline characteristics of the participants.

Characteristic	Data
Participants’ eyes, right, n (%)	439 (50%)
Age (years), mean ± SD	53.16 ± 17.14
Gender, male, n (%)	213 (48.5%)
Hypertension, n (%)	75 (17.1%)
Diabetes, n (%)	48 (10.9%)
OCT scan (seconds), mean ± SD	18.4 ± 0.11
AI outputting time (minutes), mean ± SD	4.01 ± 0.03

### Ophthalmologist diagnosis

Three groups of ophthalmologists were organized to perform manual annotation with the selection criteria as follows:

The junior group (OP1) consists of three resident doctors who had worked in the Department of Ophthalmology for more than 3 years. The senior group (OP2) consists of three attending ophthalmologists with more than 8 years of experience in clinical practice. The retinal specialist’s group (OP3) consists of three retinal professors who have dedicated to working in the field of retinal diseases for more than 12 years. The ophthalmologists were all from the Department of Ophthalmology of Shanghai Tenth People’s Hospital and trained together before the task. If the conclusions of three doctors in each group were inconsistent, a panel discussion and vote inside the group would be arranged to reach a consensus and generate a standard conclusion.

A manual review and final diagnosis of the 15 retinal disorders were prepared according to all the participant’s medical information, including slit-lamp examination, fundus photography, OCT images, demographic information, and systemic medical history. Doctors in each group carried out an independent diagnosis and were masked to AI diagnosis results from doctors in the same group as well as in other groups. The manual diagnosis and AI algorithm outputs were compared according to the recognized diagnostic criteria, noting that a combination of various pathologies could coexist in one participant.

### Statistical analysis

To evaluate the performance of the AI algorithm compared with three different levels of ophthalmologists, the receiver operating characteristic curve (ROC) was generated using the diagnosis results given by the retinal specialist (OP3) regarded as the reference standard. The area under ROC (AUC), sensitivity and specificity, and Youden index with 95% confidence intervals (95% CIs) were calculated. Paired comparisons were performed between the AUC of AI to OP3, junior ophthalmologist (OP1) to OP3, and senior ophthalmologist (OP2) to OP3 to assess their difference toward the reference standard. Kappa (*κ*) statistics were used to quantify and evaluate the degree of agreement between AI and three different level group ophthalmologists (OP1, OP2, and OP3). To transfer the AI grading score to a binary decision for analysis, the cut-off value was set at 0.7 based on the pre-setting data during model establishment, which means that a value equal to or larger than 0.7 in AI calculated results as positive, while less than 0.7 is recognized as negative. The threshold value 0.7 was selected on the validation set, which means that the AI gained the maximum value of the F1 score on the validation set when the threshold value was set at 0.7. The F1 score is an index that could balance the false detection rate and missed detection rate, and it is a commonly used index for selecting the threshold of the algorithm model. A *p*-value < 0.05 was considered statistically significant. All data generated were analyzed using SPSS version 21.0 (IBM Inc., Chicago, Illinois, United States).

## Results

From September to December 2021, 477 subjects (954 eyes) from four local communities mentioned previously participated in the study. However, after image quality control, only 878 eyes (from 439 subjects) were finally enrolled, while 76 eyes were excluded due to poor OCT image quality, mainly due to small pupils, keratoleukoma, cataracts, vitreous opacity, *etc.* The 439 subjects consisted of 213 males and 226 females, aging from 34 to 72 (53.16 ± 17.14), among whom 75 (17.1%) had a history of hypertension and 48 (10.9%) had a history of diabetes mellitus. The demographic information, average time for the OCT scan, and AI output are presented in Table 1.

The overall ROC curve comparison between AI diagnosis and the group of retinal specialists (OP3) represented a large AUC (0.891–0.997), high sensitivity (87.65–100%), and high specificity (80.12–99.41%). Since no case of RD was detected in the whole participants’ screening either by doctors or by AI software, the data related to RD were unavailable beyond any comparison or discussion. Among the rest 14 retinal disorders, the best performance was revealed in the diagnosis of CME and retinal hemorrhage, with the AUC, sensitivity, and specificity at 0.997, 96.43%, and 98.94%, and 0.991, 100%, and 96.31%, respectively. Also, high accuracy with a relatively large AUC was acquired in the diagnosis of PED, PVD, ERM, FCE, and CNV, with the AUC, sensitivity, and specificity at 0.985, 97.62%, and 96.29% in PED; 0.973, 91.83%, and 95.60% in PVD, and 0.955, 91.84%, and 93.68% in ERM, respectively. As for the diagnosis in FCE, the AUC was 0.967 with a sensitivity of 91.18% and specificity of 99.41%, while the result was 0.983, 97.22%, and 98.34%, relatively for CNV, respectively. The lower results were generated in the detection of exudation (AUC of 0.944, sensitivity of 89.57%, and specificity of 88.99%) and retinoschisi (AUC of 0.926, sensitivity of 92.23%, and specificity of 82.32%). The lowest AUC appeared in the recognition of drusen, which still reached 0.891, with a sensitivity of 92.10% and a specificity of 80.12%.

The ROC curve comparisons between the groups of junior (OP1) or senior (OP2) ophthalmologists and AI to the group of retinal specialists (OP3) were generated and compared. The paired comparison showed that the AUC of AI–OP3 was larger than that of OP1–OP3 or OP2–OP3 with a significant difference, indicating that the AI results were much closer to OP3 taken as the golden standard, surpassing OP1 or OP2.

**FIGURE 2:**
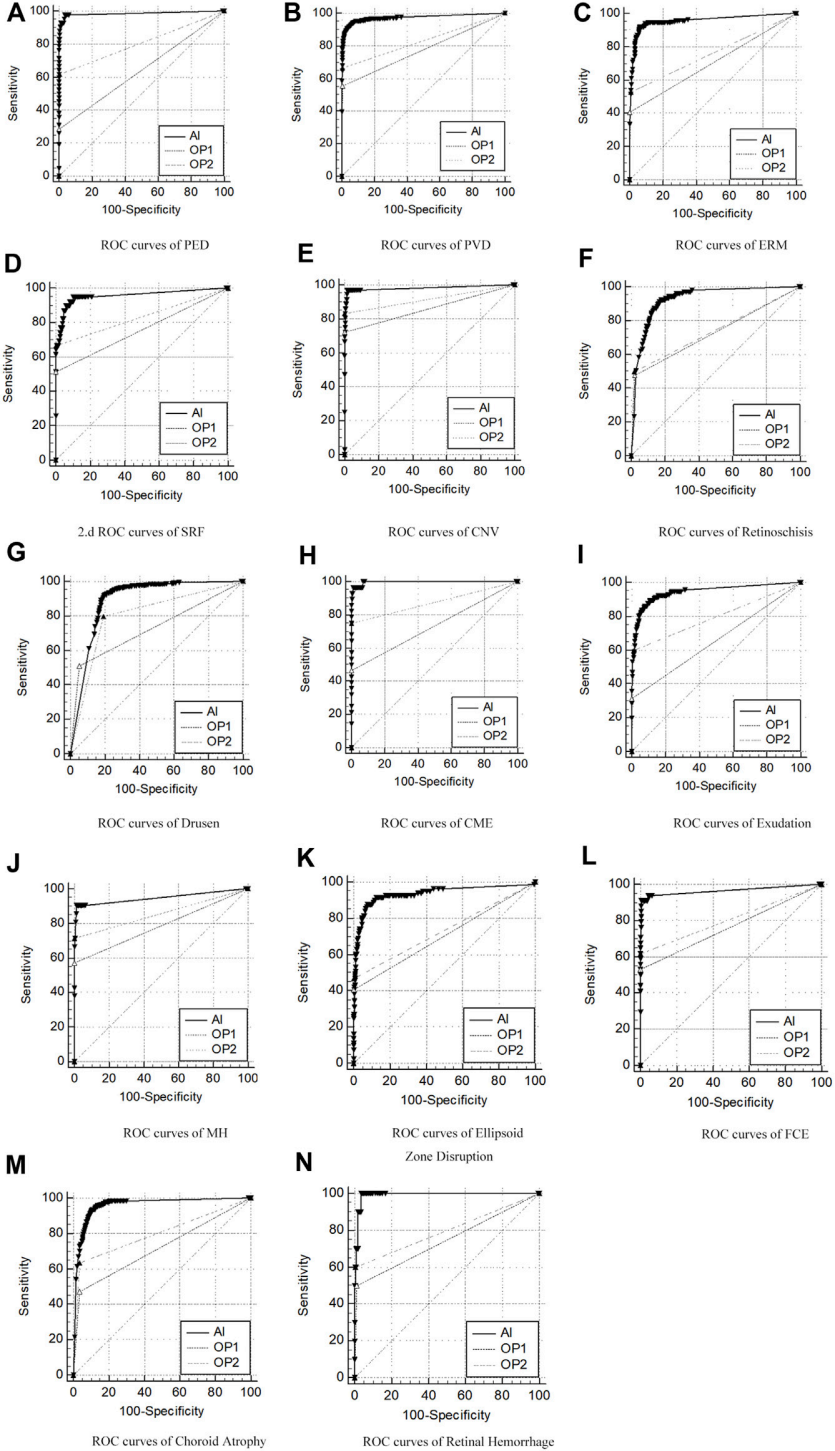
ROC curves of AI/group of junior ophthalmologists (OP1)/group of senior ophthalmologists (OP2) compared to the group of retinal specialists (OP3, as the golden standard) in diagnosis of 14 retinal disorders. **(A–N)** Pigment epithelial detachment (PED), posterior vitreous detachment (PVD), epiretinal membranes (ERMs), sub-retinal fluid (SRF), choroidal neovascularization (CNV), drusen, retinoschisis, cystoid macular edema (CME), exudation, macular hole (MH), ellipsoid zone disruption, focal choroidal excavation (FCE), choroid atrophy, and retinal hemorrhage.

**FIGURE 3:**
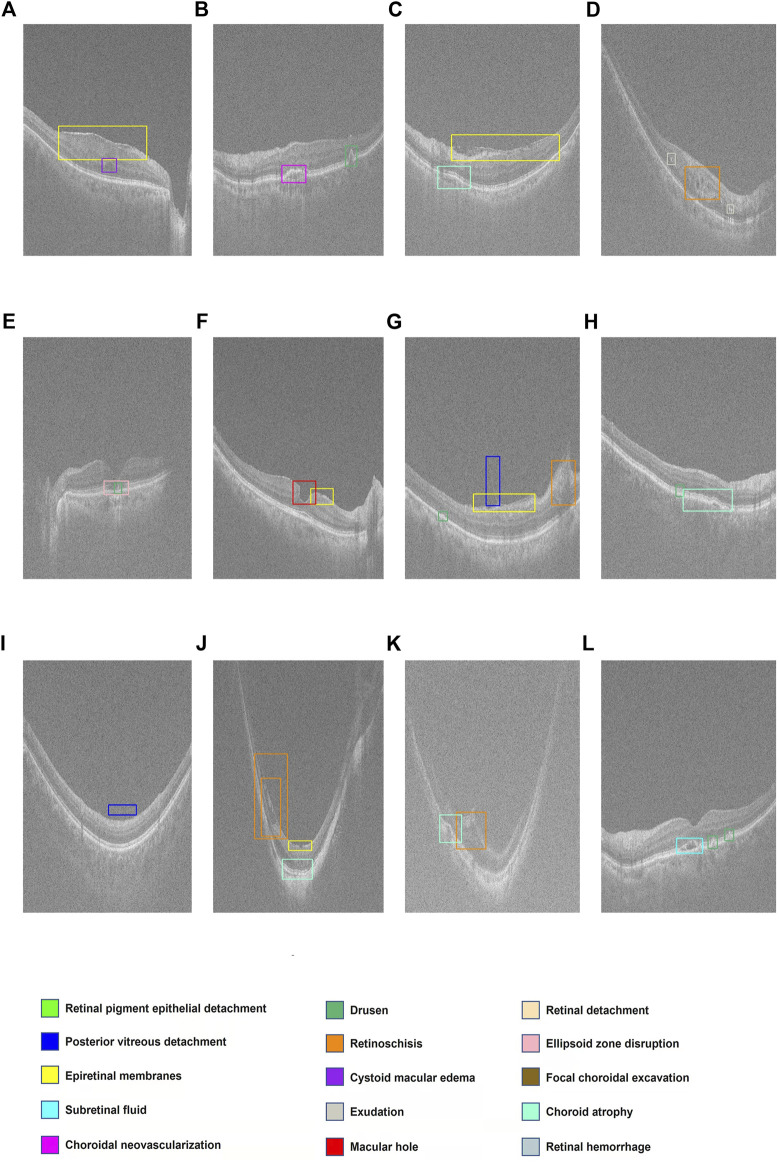
Display of multiple retinal disorders and their locations. **(A–L)** Fifteen categories of retinal disorders are marked by rectangles with specific colors on OCT images.

The ROC curves for 14 retinal disorders are shown in Figure 2, and comparisons of AUC, Youden index, specificity, and sensitivity with 95% CI are shown in Table 2. At the same time, representing images with lesions labeled at the specific location of the retina are illustrated in Figure 3.

**TABLE 2:** AUC (with 95% CI), Youden index, specificity, and sensitivity in ROC of AI/group of junior ophthalmologists (OP1)/group of senior ophthalmologists (OP2) compared to the group of retinal specialists (OP3, as the golden standard).

	AI vs. OP3							OP1 vs. OP3				OP2 vs. OP3				*p* (AUC, AI–OP3 vs. OP1–OP3)	*p* (AUC, AI–OP3 vs. OP2–OP3)
	AUC	95% CI	*p*	Youden index	Sensitivity (%)	Specificity (%)		AUC	95% CI	*p*		AUC	95% CI	*p*			
PED	0.985	0.974–0.992	<0.0001	0.9391	97.62	96.29		0.643	0.610–0.675	0.0001		0.808	0.780–0.833	<0.0001		<0.0001	<0.0001
PVD	0.973	0.960–0.983	<0.0001	0.8743	91.83	95.60		0.775	0.745–0.802	<0.0001		0.830	0.804–0.855	<0.0001		<0.0001	<0.0001
ERM	0.955	0.940–0.968	<0.0001	0.8552	91.84	93.68		0.704	0.673–0.734	<0.0001		0.759	0.730–0.787	<0.0001		<0.0001	<0.0001
SRF	0.956	0.940–0.968	<0.0001	0.8450	94.87	89.63		0.756	0.727–0.784	<0.0001		0.833	0.807–0.857	<0.0001		<0.0001	= 0.0003
CNV	0.983	0.972–0.990	<0.0001	0.9556	97.22	98.34		0.860	0.835–0.882	<0.0001		0.917	0.896–0.934	<0.0001		= 0.0007	= 0.0227
Retinoschisis	0.926	0.906–0.942	<0.0001	0.7456	92.23	82.32		0.727	0.696–0.756	<0.0001		0.739	0.708–0.767	<0.0001		<0.0001	<0.0001
Drusen	0.891	0.868–0.911	<0.0001	0.7222	92.10	80.12		0.728	0.698–0.758	<0.0001		0.803	0.775–0.828	<0.0001		<0.0001	<0.0001
CME	0.997	0.991–0.999	<0.0001	0.9537	96.43	98.94		0.732	0.702–0.761	<0.0001		0.875	0.851–0.896	<0.0001		<0.0001	= 0.0028
Exudation	0.944	0.927–0.959	<0.0001	0.7856	89.57	88.99		0.657	0.624–0.688	<0.0001		0.793	0.764–0.819	<0.0001		<0.0001	<0.0001
MH	0.948	0.931–0.962	<0.0001	0.8931	90.48	98.83		0.785	0.756–0.811	<0.0001		0.857	0.832–0.880	<0.0001		= 0.0020	= 0.0376
Ellipsoid zone disruption	0.935	0.916–0.950	<0.0001	0.8000	87.65	92.35		0.704	0.672–0.734	<0.0001		0.734	0.703–0.763	<0.0001		<0.0001	<0.0001
FCE	0.967	0.953–0.978	<0.0001	0.9058	91.18	99.41		0.764	0.735–0.792	<0.0001		0.808	0.781–0.834	<0.0001		<0.0001	= 0.0001
Choroid atrophy	0.959	0.943–0.971	<0.0001	0.8288	93.62	89.26		0.718	0.687–0.748	<0.0001		0.799	0.771–0.825	<0.0001		<0.0001	<0.0001
Retinal hemorrhage	0.991	0.982–0.996	<0.0001	0.9631	100.00	96.31		0.744	0.714–0.773	0.0034		0.798	0.770–0.824	0.0003		= 0.0022	= 0.0136

The results of the kappa analysis are shown in Table 3. The consistency between AI and retinal specialists was relatively high, with the average value of 0.731, while the average value was 0.579 between AI and OP1 and 0.707 between AI and OP2.

**TABLE 3:** Kappa analysis results.

Diagnosis	Kappa value		
	AI vs. OP1	AI vs. OP2	AI vs. OP3
PED	0.608	0.816	0.774
PVD	0.657	0.763	0.873
ERM	0.452	0.584	0.834
SRF	0.440	0.539	0.580
CNV	0.824	0.860	0.843
Drusen	0.383	0.751	0.720
Retinoschisis	0.424	0.426	0.520
CME	0.809	0.847	0.803
Exudation	0.373	0.616	0.709
MH	0.555	0.630	0.709
RD	NA	NA	NA
Ellipsoid zone disruption	0.627	0.669	0.669
FCE	0.767	0.803	0.807
CA	0.553	0.690	0.792
RH	0.635	0.899	0.595
Average	0.579	0.707	0.731

## Discussion

To relieve the conflict between the enormous demand for early screening of multiple retinal diseases with rising prevalence and the lack of human medical resources, the application of AI algorithms, especially the DL models as auxiliary tools for diagnosis provided a feasible and promising solution (He et al., 2020; Xie et al., 2020; Cen et al., 2021). Taking the retinal OCT image rather than fundus photography only as the diagnosis reference proof was a new trend in this field (Treder et al., 2018; Sogawa et al., 2020; Wang et al., 2020). However, most current software was designed to detect one single category of retinal disease, which hindered its application in real-world practice. Our study utilized an AI algorithm that can recognize 15 retinal disorders at one time with features extracted from OCT images and evaluated its accuracy and feasibility in community screening.

In this study, the demographic information showed equivalence in gender but possessed a relatively elder population age, which conformed to the actual characteristic of the four communities involved. The prevalence of hypertension (17%) and diabetes mellitus (11%) among our participants was similar to that of the general population (Wang et al., 2018; Sun et al., 2022). Except for the retinal detachment with no case occurred in the study, the incidence rate of the rest 14 retinal disorders was close to the natural scale in the general population (Hong et al., 2013; Mitchell et al., 2018; Wang and Lo, 2018; Wolfram et al., 2019; Li et al., 2022b).

According to our data, the overall ROC curve comparison between AI diagnosis and the group of retinal specialists (OP3) exhibited large AUC (0.891–0.997), high sensitivity (87.65–100%), and high specificity (80.12–99.41%). Also, the AUC of AI–OP3 was larger than that of OP1–OP3 or OP2–OP3 with a significant difference, while the average value generated from kappa analysis representing the consistency between AI and retinal specialists (OP3) was larger than AI–OP1 and AI–OP2, which in all supported that AI results were much closer to OP3 as the golden standard, exceeding the performance of OP1 or OP2, and certified the accuracy of the AI auto-detection system we utilized.

Compared to the function of other AI software developed for a specific category of retinal disorders (AMD, DR, or ERM solely) in previous studies, our system also demonstrated equal or even better performance in the corresponding specific disease. As for the detection of AMD, one of the leading causes of visual impairment in elderly patients, several deep-learning algorithms have been developed to recognize relative lesions and perform machine discrimination. Treder et al. (2018) developed an AI algorithm based on an open-source multi-layer deep CNN model to diagnose AMD and the generated sensitivity, specificity, and accuracy were 100%, 92%, and 96%, respectively. However, the size of the test was relatively small (*n* = 50). The DL model of Lee et al. (2017) achieved an area under the ROC curve of 92.78% with an accuracy of 87.63% at the image level, while at the patient level, the data were 97.45% and 93.69%, respectively. However, neither the classifiers could detect the specified lesion nor the position of AMD, which was fulfilled in our algorithm (Lee et al., 2017; Treder et al., 2018). Elsharkawy et al. (2021) reviewed several studies using DL models for AMD diagnosis in a qualitative and quantitative manner and found that despite the positive results generated from different algorithms, most of them could not identify or grade each type of AMD. Also, the majority of the testing was conducted on preselected individuals’ sample only, rather than real-world validation in our study. Mantel et al. (2021) developed a DL algorithm for the automated identification, localization, and volume measurement of exudative manifestations of intraretinal fluid (IRF), sub-retinal fluid (SRF), and pigment epithelium detachment (PED) in neovascular age-related macular degeneration (nAMD). The results showed that the AUC, sensitivity, and specificity were 0.97, 0.95, and 0.99, respectively, with accurate measurement of the volumes, despite a limited number of included OCT volumes, advancing the aspect of AI from quantitation to quantification.

As for the automated diagnosis of DR, the prevalence of which was ascending worldwide and causing a visual loss for a large population, and previous studies were carried out using fundus photographs as image sources. In contrast, recent studies were conducted utilizing OCT images (Sandhu et al., 2018; Sandhu et al., 2020; Lakshminarayanan et al., 2021). Lakshminarayanan et al. (2021) reviewed different ML models on DR diagnosis published within 6 years (2016–2021) and concluded that although some of the recent CNN-based models exhibited high performance in terms of the standard metrics, the lack of validation for real-life clinical applications remained as defects, with difficulty in detecting specific lesion like exudation and microaneurysms.

Previous studies also involved auto-detection of myopic macular diseases using AI algorithm due to the rising prevalence of myopia and multiple vision-threatening retinal damages (Sogawa et al., 2020; Choi et al., 2021; Ye et al., 2021; Li et al., 2022a). Ye et al. (2021) engineered the deep-learning (DL) model to identify myopic maculopathy, including macular choroidal thinning, macular Bruch membrane (BM) defects, sub-retinal hyper-reflective material (SHRM), myopic traction maculopathy (MTM), and dome-shaped macula (DSM), and the result showed that the AUC was 0.927–0.974 for five myopic maculopathies. Choi et al. (2021) trained and validated three DL models to identify myopia and generated a result of the absolute agreement with retina specialists which was 99.11%. However, the specific lesion associated with myopia could not be detected, and validation with an external dataset was needed. Li et al. (2022a) developed four independent CNN models to identify retinoschisis, macular hole, retinal detachment, and pathological myopic choroidal neovascularization and acquired satisfactory results with a high AUC for all conditions (0.961–0.999), revealing that the sensitivity and specificity of the AI system were equal to or even better than those of retina specialists.

Among the literature, we reviewed another two specific retinal disorders: ERM and PED (Sun et al., 2016; Lo et al., 2020). Lo et al. (2020) proposed a deep-learning model to identify the epiretinal membrane (ERM) in OCT. Also, the results showed that the diagnostic accuracy was 98.1% and the AUC was 0.999, implying that the model’s performance was slightly better than the average non-retinal specialized ophthalmologists. Sun et al. (2016) proposed an automated framework to segment serous PED in SD-OCT images. The average true-positive volume fraction (TPVF), false-positive volume fraction (FPVF), dice similarity coefficient (DSC), and positive predictive value (PPV) were calculated as 90.08%, 0.22%, 91.20%, and 92.62%, respectively. However, the test dataset consisted of only 25 patients.

No literature was found concerning AI auto-detection based on OCT images of the following specific retinal disorders: ellipsoid zone disruption, FCE, PVD, and RD.

Through the aforementioned literature review, we saw a different performance of various AI models, as well as compared our results. The underlying reason may include: 1) different models and architecture; 2) variant resources and sizes of the dataset for training and testing; 3) inconsistent standards for lesion labeling, as well as the difference in definition and classification of a specific disease; 4) different procedures of image quality control, as the quality of an image may affect the results of AI output; and 5) different methodology of evaluation, as with image view (only judge the image) or patient view (with consideration to other clinical information.)

A common disadvantage of the aforementioned studies was that their AI models focused on only one specific retinal disorder leaving others unable to be recognized, which reduced their feasibility and availability in real-world practice as community screening (Kuwayama et al., 2019; Wang et al., 2020; Guo et al., 2021; Liu et al., 2022). Our AI model could identify multiple retinal disorders simultaneously in single detection, which was more appropriate for the scene of community screening with the unpredicted situations and comprehensive diseases and saved more time and occupied fewer human resources. In addition to the AUC and specificity, our results revealed a relatively high sensitivity, which ensured its potency as a screening tool in the early stage. In the procedure of the OCT scan, the average examination time was 18.4 s for one eye and the average output time of the AI report was 4 min. The high accuracy and efficiency were proposed due to 1) the strategy in architecture and model establishment, as well as the online hard example mining mechanism utilized to improve the convergence speed due to the unbalanced ratio between the foreground and background; 2) accumulated experience and advanced technology in OCT image analysis with AI algorithms (Sun et al., 2016; Zhu et al., 2017; Shi et al., 2019; Shi et al., 2021; Wang et al., 2022); 3) the integrated design of the OCT instrument and AI algorithm; 4) the high performance of the OCT instrument with a maximum A scan speed at 45,000 times per second as well as the high resolution of the images. The aforementioned issues all guaranteed the accuracy and speed of the community screening work.

However, there are still some limitations to our study. First, the images were acquired from one type of the OCT instrument, and the procedure was carried out in one district by one single medical center. To certify the accuracy of the AI system about images from other OCT instruments and the participants from other communities with other medical centers, we will further promote the study. The second issue was that the detection of RD was not verified due to the limitation of the participants in our study. Further evaluation may be performed with specified patients. Finally, for some retinal disorders, the performance of specificity and sensitivity still needs to be improved with the further imperfection of the algorithm.

## Conclusion

To the best of our knowledge, this was the first study carried out in real-world community screening utilizing the SD-OCT and integrated AI algorithm to auto-detect 15 different retinal disorders simultaneously, with its accuracy compared to three different levels of ophthalmologists judging from the patient aspects. Positive results revealed that the accuracy of AI was close to that of the retinal specialist, surpassing that of junior and senior ophthalmologists, indicating a promising prospect of the application of the OCT instrument and AI software in clinical practice and community screening.

## Data Availability

The original contributions presented in the study are included in the article/Supplementary Material; further inquiries can be directed to the corresponding author/s.
