# Supplementary material for: Accuracy and feasibility with AI-assisted OCT in retinal disorder community screening
Source: Front Cell Dev Biol. 2022 Nov 3;10:1053483. doi: 10.3389/fcell.2022.1053483 (PMC9670537; doi:10.3389/fcell.2022.1053483)
Supplement: Supplementary file 1 [file Table1.DOCX]

Supplement Table 1. The distribution of the datasets.

| Normal | 363 |
| --- | --- |
| PED | 30 |
| PVD | 451 |
| ERM | 188 |
| SRF | 93 |
| CNV | 87 |
| Drusen | 581 |
| Retinoschisis | 146 |
| CME | 100 |
| Exudation | 206 |
| MH | 27 |
| RD | 12 |
| Ellipsoid Zone Disruption | 174 |
| FCE | 23 |
| Choroid atrophy | 193 |
| Hemorrhage | 59 |
| Total | 1508 |
